# Supplementary material for: Perceptions on the Implementation of a School Nursing Pilot Programme in the Canary Islands
Source: Nurs Rep. 2025 Jan 31;15(2):48. doi: 10.3390/nursrep15020048 (PMC11858167; doi:10.3390/nursrep15020048)
Supplement: Supplementary file 1 [file nursrep-15-00048-s001.zip › Supplementary file 1.docx]

**Supplementary Table S1. Number of co-occurrences between sub-themes among nurses**

|  | Nurses-Workload | Nurses-School Nurses’ Experiences | Nurses-Career Opportunities | Nurses-The School Nurse Profile | School Nursing Project-Suggested Improvements | School Nursing Project-Identified Weaknesses | School Nursing Project-Time Management | School Nursing Project-Improvement Needs |
| --- | --- | --- | --- | --- | --- | --- | --- | --- |
| Nurses-Workload | 0 | 17 | 19 | 0 | 8 | 1 | 0 | 17 |
| Nurses-School Nurses’ Experiences | 17 | 0 | 52 | 9 | 12 | 1 | 2 | 27 |
| Nurses-Career Opportunities | 19 | 52 | 0 | 1 | 12 | 1 | 0 | 28 |
| Nurses-The School Nurse Profile | 0 | 9 | 1 | 0 | 16 | 15 | 80 | 8 |
| School Nursing Project-Suggested Improvements | 8 | 12 | 12 | 16 | 0 | 5 | 15 | 18 |
| School Nursing Project-Identified Weaknesses | 1 | 1 | 1 | 15 | 5 | 0 | 15 | 6 |
| School Nursing Project-Time Management | 0 | 2 | 0 | 80 | 15 | 15 | 0 | 7 |
| School Nursing Project-Improvement Needs | 17 | 27 | 28 | 8 | 18 | 6 | 7 | 0 |
