# Supplementary material for: Perceptions on the Implementation of a School Nursing Pilot Programme in the Canary Islands
Source: Nurs Rep. 2025 Jan 31;15(2):48. doi: 10.3390/nursrep15020048 (PMC11858167; doi:10.3390/nursrep15020048)
Supplement: Supplementary file 1 [file nursrep-15-00048-s001.zip › Supplementary file 2.docx]

**Supplementary Table S2. Number of co-occurrences between sub-themes among teachers**

|  | Nurses-Approach to School Nursing | Nurses-Importance of the Presence of School Nurses | School Nursing Project-Expectations | School Nursing Project-Improving Children’s Health | School Nursing Project-School Health | School Nursing Project-Experiences from the Pilot Programme |
| --- | --- | --- | --- | --- | --- | --- |
| Nurses-Approach to School Nursing | 0 | 1 | 0 | 12 | 1 | 19 |
| Nurses-Importance of the Presence of School Nurses | 1 | 0 | 0 | 1 | 27 | 1 |
| School Nursing Project-Expectations | 0 | 0 | 0 | 2 | 23 | 0 |
| School Nursing Project-Improving Children’s Health | 12 | 1 | 2 | 0 | 4 | 36 |
| School Nursing Project-School Health | 1 | 27 | 23 | 4 | 0 | 1 |
| School Nursing Project-Experiences from the Pilot Programme | 19 | 1 | 0 | 36 | 1 | 0 |
